# Tertiary syphilis mimicking metastatic rectal cancer

**DOI:** 10.1093/jscr/rjac093

**Published:** 2022-03-26

**Authors:** Matthew J Smith, Meydene Ong, Abrar Maqbool

**Affiliations:** Department of General Surgery, Griffith Base Hospital, Griffith, New South Wales, Australia; Faculty of Medicine, University of New South Wales, Sydney, New South Wales, Australia; Department of General Surgery, Griffith Base Hospital, Griffith, New South Wales, Australia; Medical Imaging Department, Prince of Wales Hospital, Sydney, New South Wales, Australia; Department of General Surgery, Griffith Base Hospital, Griffith, New South Wales, Australia

**Keywords:** tertiary syphilis, hepatic gummas, rectal cancer, liver lesions

## Abstract

Syphilis is a sexually transmitted infection caused by the bacterium *Treponema pallidum*. Tertiary syphilis, a late-stage multi-visceral complication of the disease is characterized by its diversity of clinical manifestations. Here, we present the first documented case of tertiary syphilis that clinically and radiologically mimicked primary rectal cancer with hepatic metastasis.

## INTRODUCTION

Syphilis is a sexually transmitted infection caused by the spirochete bacterium *Treponema pallidum*. Over the preceding two decades, the incidence of syphilis has been increasing in Europe and North America, whilst it remains endemic in low- and middle-income countries [1]. Men who have sex with men are at the greatest risk of primary infection, particularly in the setting of HIV co-infection [2]. Untreated, syphilis infections variably progress through several stages, termed primary, secondary and tertiary. Primary syphilis results in the formation of a chancre, a transient painless ulcer, at the site of mucosal breach by the spirochete. Secondary and tertiary syphilis may follow due to systemic dissemination of the spirochete, resulting in a vast array of cutaneous and visceral manifestations [3].

## CASE REPORT

**Figure 1:**
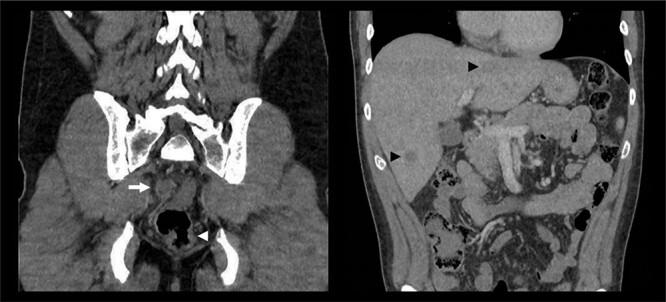
CT abdomen-pelvis with intravenous contrast demonstrating rectal wall irregularity (white arrowhead), bulky mesorectal lymphadenopathy (white arrow) and multiple hypoattenuating liver lesions (black arrow heads).

A 39-year-old man presented to the emergency department with a one-day history of right upper quadrant and several weeks of fluctuating tenesmus and diarrhoea on a background of HIV infection and previous bacterial sexually transmitted infections. The patient identified as homosexual and reported previous intravenous drug use with methamphetamine. There was no history of viral hepatitis and immunizations were up to date. Examination demonstrated a tender right upper quadrant without peritonism. Blood investigations were non-specific, showing an isolated elevation of C-reactive protein to 69 mg/l, with normal white cell count, lipase and liver function enzymes. Serological studies were negative for Chlamydia and Gonorrhoea species and confirmed an undetectable viral load of HIV RNA. Faeces and urine cultures did not isolate any organisms. A CT abdomen-pelvis with intravenous contrast was performed, demonstrating short segment irregular rectal wall thickening, bilateral mesorectal lymphadenopathy and multiple hypoattenuating liver lesions—an overall appearance highly suspicious for metastatic rectal cancer (Fig. 1).

**Figure 2:**
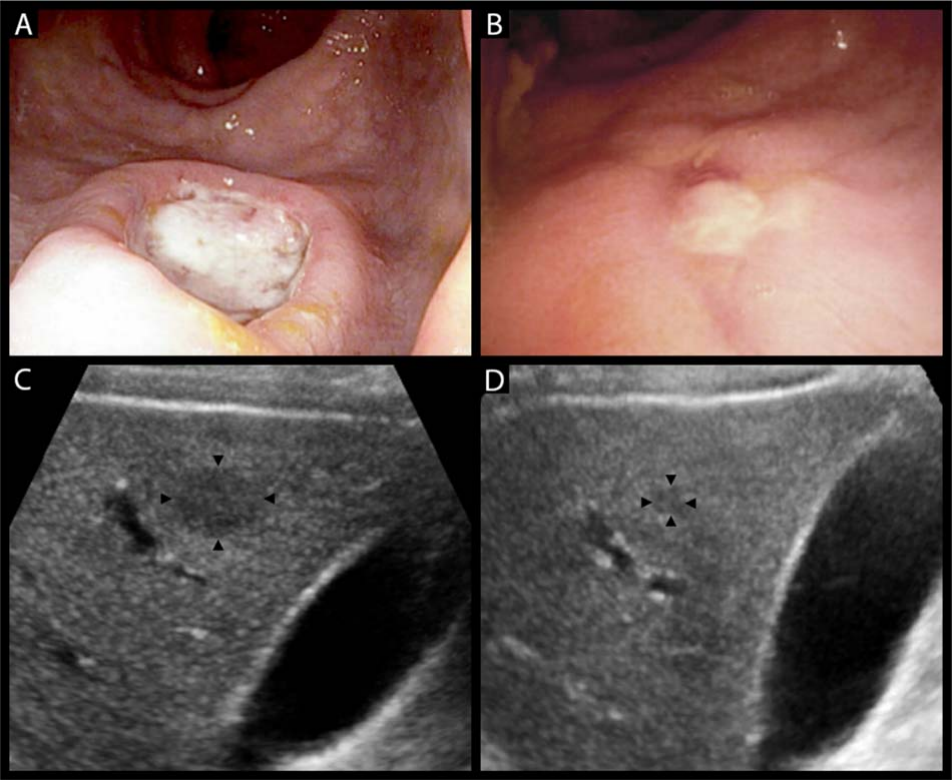
(**A**) heaped ulcer with a necrotic base in the lower rectum seen on colonoscopy, representing a syphilitic chancre. (**B**) The same lesion resolving on interval colonoscopy 4 weeks later, prior to the commencement of treatment for syphilis. (**C**) Hypoechoic liver lesion in segment 6 measuring 19 by 14 mm. (**D**) The same lesion has decreased to 6 by 5 mm at 6 weeks post-treatment.

An inpatient colonoscopy demonstrated a 2-cm ulcer with heaped margins and a necrotic base in the lower rectum that was biopsied with endoscopic forceps (Fig. 2A). A liver ultrasound was performed for further characterization of the liver lesions (Fig. 2C). The patient was discharged with resolution of his acute symptoms, for ongoing close follow-up as an outpatient. Histopathology of the rectal lesion returned as ulceration with chronic inflammation and atypical crypt epithelium, with no evident neoplastic change on immunostaining. An early interval elective colonoscopy was attended at which time the rectal ulcer had almost entirely resolved (Fig. 2B). Concurrent gastroscopy at this time was unremarkable. The patient was referred for a CT-guided liver biopsy, with a finding of scattered granulomas with extensive areas of necrosis. There were no observed neoplastic cells or microorganisms.

The case was discussed with the patient’s HIV physician who informed the team of the patient’s history of multiple episodes of syphilis infection. Following this, serological assessment with the rapid plasma reagin test returned a titre of 1:32, increased from a previous result of 1:16, confirming syphilis reinfection. The gross and histological appearances of the rectal lesion were considered consistent with a syphilitic chancre, with the liver lesions presumed to be visceral gummata—granulomatous lesions of tertiary syphilis. The patient was commenced on a three-dose course of intramuscular benzathine penicillin G. A repeat liver ultrasound at 6 weeks post-treatment demonstrated a significant reduction in the size of a dominant lesion in the right anterior liver section, with no other appreciable lesions (Fig. 2D). The patient awaits repeat syphilis serological testing and surveillance liver ultrasound to confirm complete clearance.

## DISCUSSION

Hepatic gummata and visceral gummata more broadly were a previously well-recognized consequence of prolonged syphilis infection, achieving relative obscurity following the advent of penicillin antibiotics in the mid-20th century [3]. Hepar lobatum, a lobulated distortion of the liver secondary to multiple cicatrising lesions, was previously used exclusively to describe the gummatous liver, although has since been co-opted to describe similar changes observed in the setting of metastatic adenocarcinoma [4].

Tertiary syphilis mimicking hepatic metastasis is seldom reported in the literature. In 2014 Gaslightwala *et al.* reported on a patient with severe weight loss and constitutional symptoms with PET-CT findings of FDG-avid liver lesions suggestive of metastases. Following two negative biopsies and the development of ocular and cutaneous manifestations of tertiary syphilis, the diagnosis was suspected and confirmed with immunohistochemical staining [5]. Shim reported on a case of liver gummata mimicking metastatic recurrence of a primary peritoneal serous carcinoma identified on surveillance imaging. The diagnosis was suspected following serial histological findings of necrotising granulomata without evidence of dysplasia or neoplasia [6].

These case reports highlight the challenges of establishing tertiary syphilis as the aetiology of multifocal liver lesions. CT characteristics of hepatic gummata include hypoattenation with peripheral enhancement and central necrosis or scaring–findings that carry a range of differentials including primary or metastatic malignancy [7]. If biopsy is performed, the classical findings of necrosis and granuloma formation are not disease-specific and do not exclude malignancy. Treponema species will not be identified on routine microbiological staining such as the Periodic acid-Schiff, Ziehl-Neelsen and methenamine silver stains, as were performed in our case. In addition, syphilis-specific direct fluorescent antibody staining and PCR are not widely available and are associated with low sensitivity due to the spirochete-deplete nature of gummata [8]. As such, the diagnosis often relies on clinical suspicion, serological testing and resolution following empirical treatment. Tertiary syphilis can be effectively treated with three doses of intramuscular penicillin, to which resistance has never been demonstrated [8].

This is the first reported case of syphilis masquerading as both a primary rectal malignancy and hepatic metastases, an excellent demonstration of the legion manifestations of syphilis, strengthening its claim to the title of ‘The Great Mimicker’. This once prevalent disease is anticipated to see renewed clinical significance with its rapidly increasing incidence, and as such, warrants increased suspicion in the at-risk surgical patient.
